# CaP-coated Zn-Mn-Li alloys regulate osseointegration via influencing macrophage polarization in the osteogenic environment

**DOI:** 10.1093/rb/rbad051

**Published:** 2023-05-09

**Authors:** Huifen Qiang, Caiyao Hou, Yujue Zhang, Xin Luo, Jun Li, Chunxiu Meng, Kun Liu, Zhaoyong Lv, Ximeng Chen, Fengzhen Liu

**Affiliations:** Department of Materials Science and Engineering, Liaocheng University, Liaocheng, China; Department of Materials Science and Engineering, Liaocheng University, Liaocheng, China; Liaocheng People’s Hospital, Liaocheng Dongchangfu People’s Hospital, Liaocheng 252000, China; School of Materials Science and Engineering, University of Science and Technology, Beijing 100083, China; Liaocheng People’s Hospital, Liaocheng Dongchangfu People’s Hospital, Liaocheng 252000, China; Liaocheng People’s Hospital, Liaocheng Dongchangfu People’s Hospital, Liaocheng 252000, China; Liaocheng People’s Hospital, Liaocheng Dongchangfu People’s Hospital, Liaocheng 252000, China; Liaocheng People’s Hospital, Liaocheng Dongchangfu People’s Hospital, Liaocheng 252000, China; Liaocheng People’s Hospital, Liaocheng Dongchangfu People’s Hospital, Liaocheng 252000, China; Liaocheng People’s Hospital, Liaocheng Dongchangfu People’s Hospital, Liaocheng 252000, China; Department of Materials Science and Engineering, Liaocheng University, Liaocheng, China; Liaocheng People’s Hospital, Liaocheng Dongchangfu People’s Hospital, Liaocheng 252000, China

**Keywords:** Zn-Mn-Li alloys, CaP coatings, macrophages polarization, osteointegration, inflammation

## Abstract

Immune response is an important factor in determining the fate of bone replacement materials, in which macrophages play an important role. It is a new idea to design biomaterials with immunomodulatory function to reduce inflammation and promote bone integration by regulating macrophages polarization. In this work, the immunomodulatory properties of CaP Zn-Mn-Li alloys and the specific mechanism of action were investigated. We found that the CaP Zn0.8Mn0.1Li alloy promoted the polarization of macrophages toward M2 and reduced inflammation, which could effectively upregulate osteogenesis-related factors and promote new bone formation, indicating the important role of macrophages polarization in biomaterial induction of osteogenesis. *In vivo* studies further demonstrated that CaP Zn0.8Mn0.1Li alloy could stimulate osteogenesis better than other Zn-Mn-Li alloys implantations by regulating macrophages polarization and reducing inflammation. In addition, transcriptome results showed that CaP Zn0.8Mn0.1Li played an important regulatory role in the life process of macrophages, activating Toll-like receptor signaling pathway, which participated in the activation and attenuation of inflammation, and accelerated bone integration. Thus, by preparing CaP coatings on the surface of Zn-Mn-Li alloys and combining the bioactive ingredient with controlled release, the biomaterial will be imbibed with beneficial immunomodulatory properties that promote bone integration.

## Graphical abstract

**Figure:**
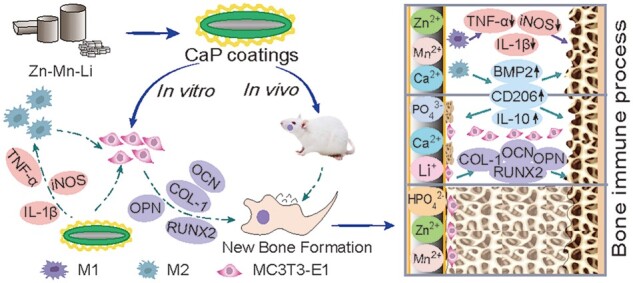

## Introduction

Zn alloys implanted biomaterials have good biocompatibility and mechanical properties and play an important role in medical degradable implant materials [1, 2]. The human body is a complex and changing environment, and the implantation of materials can produce a range of biological reactions [3]. More and more evidence suggested that the early inflammatory response of immune cells (neutrophils, lymphocytes and macrophages) to the implant surface determined the osteogenic effect after implantation. Among them, different polarization types of macrophages play an important role in bone immune regulation [4]. Advances in materials biology had shown that manipulating the surface properties of implantable materials, such as surface morphology and composition, can regulate a variety of cell behaviors, including cell viability and cell function [5, 6]. Studies on bone immunology had demonstrated that the immune and skeletal systems shared some cytokines, receptors and transcription factors that were involved in bone reconstruction [7]. It had been proved that Mg-Si-Ca alloys can activate mononuclear macrophages cell lines, forming an immune microenvironment conducive to recruitment of mesenchymal stem cells (MSCs) and initiation of osteogenic differentiation [8]. The immune microenvironment generated by a custom-made honeycomb TiO_2_ structure on a titanium (Ti) substrate was also beneficial for stimulating osteogenic differentiation of murine MSCs *in vitro* and subsequently promoting implant bone integration *in vivo* [9]. These studies suggested that surface modification methods used to promote bone integration should focus not only on osteogenesis but also on immune regulation to establish an optimal environment for promoting bone repair. Signals from different implantations surfaces may trigger a switch in macrophages phenotypes associated with immune responses and subsequently trigger a bone-enabling signal. Therefore, the immunomodulatory capacity of bone will be an important index to evaluate the surface modification of Zn-Mn-Li scaffolds.

Macrophages play a key role in immune responses and are responsible for a variety of bone immune responses [10, 11]. Macrophages participate in bone repair and become the target cells for primary bone therapy, which is related to the polarization of macrophages. In the early stage of implantation, as a response to tissue injury, macrophages polarize to M1 pro-inflammatory phenotype and secrete cytokines and chemokines, including TNF-α, IL-6, IL-1β, etc., to recruit additional immune cells. A few days later, macrophages polarize to M2 and secrete different cytokine environments, such as TGF-β to promote healing. Overexpression of either phenotype can lead to scar formation, and a delicate balance between M1 and M2 polarization is necessary to promote bone remodeling [12, 13]. An excessive inflammatory response may create a fibrous coating around the implantations, inhibiting the production of osteogenic factors, which can inhibit bone integration [14]. During bone regeneration and repair, an appropriate immune response is essential, such as maintaining the elastic modulus of the bone, which can effectively prevent fractures. Macrophages could promote rapid mineralization of osteoblasts, and the depletion of macrophages will lead to loss of bone formation [15]. Macrophages have good plasticity and become the target of immune regulation. In view of the role of macrophages in bone formation and dynamic balance, the response of macrophages to biomaterials can be used to evaluate the bone immunomodulatory properties of materials.

The surface properties of biomaterials affect the surrounding cells [2]. Calcium (Ca) and phosphorus (P) have been shown to regulate macrophages polarization toward M2, reducing inflammatory response and promoting osteogenesis. Phosphate chemical conversion technology has the advantages of low cost, simple operation, suitable for irregular surface and little influence on mechanical properties of the matrix, which is an excellent technology for surface modification of Zn alloys. And elements with bone regeneration, antibacterial and immunomodulatory functions can be selected to obtain morphological characteristics with related biological functions and conducive to cell adhesion. In addition, the coatings deposited by phosphate chemical conversion technology have better crystallinity and stability, which can provide good protection for Zn-Mn-Li alloys. On the one hand, it can reduce the ion release rate and toxic reaction, on the other hand, it can regulate the immune response to ensure the long-term stability of implants during service [16–18]. In our previous studies, high-performance Zn-Mn-Li alloys and bioactive CaP coatings on their surface were prepared and their cytocompatibility evaluation was discussed. After the addition of Mn/Li elements, the second phase of MnZn_13_ and LiZn_4_ appeared, the grain was refined, the hardness was increased, the corrosion became uniform and no serious pitting and local corrosion occurred. After the CaP coatings were deposited on the Zn-Mn-Li scaffolds, the hydrophilicity and roughness of the scaffolds were obviously improved, which were more suitable for cell attachment. *In vitro* degradation experiments confirmed that Zn-Mn-Li alloys scaffolds coated with CaP coatings had a slow degradation rate, and metal ions could be released continuously and slowly to maintain appropriate metal ion concentration in the surrounding area, thus reduced toxicity and enhanced the proliferation and adhesion of MC3T3-E1 cells. Previous studies had shown that CaP coatings had good physicochemical properties and osteogenic potential [2]. However, in order to promote the clinical application of CaP Zn-Mn-Li scaffolds, further characterization of bone immunomodulatory properties is required.

In this study, the bone immunomodulatory capacity of CaP Zn-Mn-Li scaffolds was evaluated. At the same time, the potential mechanism of CaP Zn-Mn-Li scaffolds regulating macrophages polarization to promote bone repair was explored. Our study aimed to clarify the application of CaP Zn-Mn-Li implants in bone immune regulation.

## Materials and methods

### The sample processing

Under the protection of Ar atmosphere, Zn, Mn and Li metal powders were put into the ZG-0.01 vacuum induction furnace, heated at 500°C for 40 min, poured into graphite mold to obtain cylindrical ingot, and then homogenized at 250°C for 2 h and 350°C, respectively, to obtain Zn-Mn-Li alloys. The alloys were cut into a 10 mm diameter and 1 mm thickness round sheet, which were then polished with 240–5000 SiC sandpaper to obtain a smooth surface.

The CaP coatings were prepared on the surface of Zn-Mn-Li alloys by phosphate chemical conversion technology. After acid etching, the samples were connected with pure iron clips, immersed in phosphate chemical conversion solution with Ca/P ratio of 1.67 and pH2.5–3.5, heated at 70°C for 60 min, and ultrasonic for 60 min to obtain CaP Zn-Mn-Li alloys scaffolds. All samples were sterilized in 75% ethanol solution for 20 min and exposed to UV radiation for 20 min.

### Cell culture

RAW264.7 cells were cultured in DMEM supplemented with 10% FBS and 1% penicillin-streptomycin. MC3T3-E1 cells were cultured in α-MEM containing 10% FBS, 1% L-glutamine and 1% penicillin-streptomycin.

### Macrophages proliferation and cytocompatibility

RAW264.7 cells were inoculated on the surface of Zn-Mn-Li scaffolds and cultured for 1, 3 and 7 days, 10% CCK-8 reagent was added and incubated at 37°C for 3 h. The cell proliferation capacity was detected by microtablet plate reader (Thermo, USA).

For the cell adhesion test, RAW264.7 cells were fixed with 4% paraformaldehyde, dehydrated with gradient ethanol solution, dried at room temperature and then FE-SEM was used to observe the morphology of the cells. DAPI nuclear staining was used to determine the adhesion number of macrophages. The cell viability was measured using the AM/PI^®^viability/cytotoxicity kit (Invitrogen).

For the morphology analysis of macrophages skeleton, the fixed cells were permeabilized using 0.1% Triton. Then, podophyllotoxin dye and 1% bovine serum albumin were stained for 30 min in the dark. Finally, DAPI was added for 30 s to stain the nuclei. Images were captured by fluorescence microscope (Nikon, Japan).

### Macrophages polarization

After RAW264.7 cells were cultured on the Zn-Mn-Li alloys for 1, 3 and 7 days, digested with trypsin, fixed for 20 min, then suspended with washing buffer, and added with 2 μl FITC-conjugated CD86 and APC-conjugated CD206 antibodies. Incubated away from light for 30 min. The staining was analyzed by flow cytometry (BD FACSArIa 111).

To further investigate the effects of the Zn-Mn-Li alloys on the polarization direction of macrophages, the expression levels of TNF-α, iNOS, IL-1β, CD206, BMP2 and IL-10 were detected by qRT–PCR. All primer sequences were shown in Table 1.

**Table 1.** Primer sequences were used in the study

Gene	Primer	Sequences (5′–3′)
TNF-α	Forward	CCCTCACACTCACAAACCAC
	Reverse	ACAAGGTACAACCCATCGGC
iNOS	Forward	CACCAAGCTGAACTTGAGCG
	Reverse	CGTGGCTTTGGGCTCCTC
IL-1β	Forward	TGCCACCTTTTGACAGTGATG
	Reverse	TGATGTGCTGCTGCGAGATT
CD206	Forward	AGACGAAATCCCTGCTACTG
	Reverse	CACCCATTCGAAGGCATTC
BMP2	Forward	TTCCATCACGAAGAAGCCGT
	Reverse	GAAACTCGTCACTGGGGACA
IL-10	Forward	GTAGAAGTGATGCCCCAGGC
	Reverse	CACCTTGGTCTTGGAGCTTATT
RUNX2	Forward	AAATGCCTCCGCTGTTATGAA
	Reverse	GCTCCGGCCCACAAATCT
OPN	Forward	ATCTCACCATTCGGATGAGTCT
	Reverse	TGTAGGGACGATTGGAGTGAAA
OCN	Forward	CCGGGAGCAGTGTGAGCTTA
	Reverse	AGGCGGTCTTCAAGCCATACT
COL-1	Forward	GCTGGAGTTTCCGTGCCT
	Reverse	GACCTCGGGGACCCATTG
Csf3	Forward	CATGAAGCTAATGGCCCTGC
	Reverse	CTGGCCTGGATCTTCCTCAC
lcn2	Forward	TCTGTCCCCACCGACCAA
	Reverse	GGAAAGATGGAGTGGCAGACA
Cx3cr1	Forward	CCATCTGCTCAGGACCTCAC
	Reverse	CACCAGACCGAACGTGAAGA
Lilra6	Forward	GAAGCCAGCAAACAAGGCTG
	Reverse	GTGTCCAGTAGTGTCCTGTCA
Gbp8	Forward	CAGAAGGCCATTGCAGAGGA
	Reverse	TCTCTCTCCTGCTCCAGCTT
GAPDH	Forward	TGACCACAGTCCATGCCATC
	Reverse	GACGGACACATTGGGGGTAG

### Preparation of Zn-Mn-Li scaffolds/macrophages conditioned medium and their effects on osteogenic differentiation of MC3T3-E1 cells

RAW264.7 cells were inoculated on Zn-Mn-Li scaffolds and the supernatant was collected after half-liquid replacement every day. Collect for 3 days and mixed with osteoblast medium in accordance with 1:3 to obtain conditioned medium (CM). The MC3T3-E1 cells were cultured in CM for 1, 3 and 7 days. Cell proliferation, cell viability and cytoskeleton were measured as previously described.

In order to investigate the effects of immune microenvironment generated by Zn-Mn-Li scaffolds on osteogenesis, MC3T3-E1 cells were stimulated with CM containing 10 nM dexamethasone, 50 μg/ml vitamin C and 10 mM glycerol phosphate.

After 7 days of culture, ALP staining was performed. Fixed with 4% paraformaldehyde, then added BCIP/NBT staining solution (Beyotime) according to the product instruction, incubated for 30 min away from light and observed under a microscope.

Alizarin red staining was performed after 14 days of culture. Fixed with 4% paraformaldehyde, dyed 10 min with alizarin red S dye (Oricell), washed with distilled water and observed under light microscope.

After 7 days of culture, the expressions of osteogenic genes (COL1, OPN, OCN and RUNX2) were further detected by qRT–PCR.

### Rat mandibular defect model

**Figure 1.**
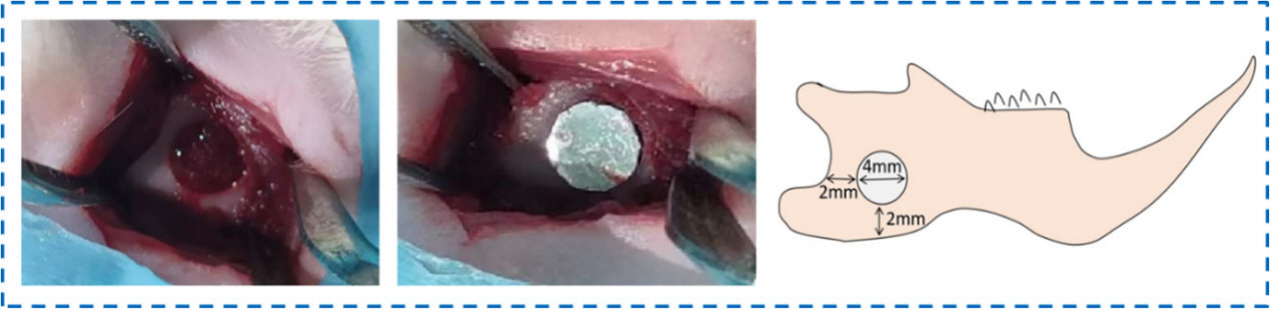
Surgical procedure and rat model of mandibular defect.

Eight-week-old rats were anesthetized with 10% chloral hydrate. The surgical procedure was shown in Fig. 1. After skin preparation and disinfection, the skin and subcutaneous tissue were dissected layer by layer along the mandibular angle of rats, and a circular bone drill with a diameter of 4 mm was used to prepare a circular penetrating bone defects at the mandibular angle of rats. After that, sterilized Zn-Mn-Li scaffolds were implanted and sutured layer by layer. At 2, 4 and 8 weeks, the rats were sacrificed, and the mandibles were removed, which were then fixed, decalcified, embedded, sectioned and dewaxed for subsequent tests.

### Imaging examination

After the mandible was removed and fixed in 4% paraformaldehyde for 24 h, the mandible specimens of each group were scanned by X-ray to observe bone formation and material degradation. Casting conditions: 63 kV, 8 mA, 0.16 s (Bruker SkyScan 2211, Belgium).

### H&E and Masson staining

After paraffin embedding, cut into 4 μm continuous slices using Leica Biosystems RM2255 (Leica, Germany). After dewaxing and hydration, hematoxylin and eosin (H&E) staining was performed, the nucleus was stained with hematoxylin, and the cellular plasma and extracellular matrix were stained with eosin after dehydration. Masson staining (Solarbio, Beijing, China) was performed as requested by the manufacturer to detect collagen deposition. The histological observation was carried out under the Axioskop 40 microscope (ZEISS, Germany). Image-Pro Plus 6.0 was used to conduct a semi-quantitative analysis of the staining results, the scale of the analysis index was converted from gray value to standard optical density value and the situation of new bone formation and inflammation regression was calculated by IOD/Area.

### RNA sequencing

Gene expression in macrophages on scaffolds was detected by RNA sequencing. Macrophages were inoculated on the scaffolds and cultured for 3 days. Then TRIzol reagent was used to collect cell RNA and the entire gene expression was examined by the Novogene Co., Ltd. The expression of related differential genes was detected by heat map and evaluated by GO analysis and KEGG pathway analysis. qRT–PCR was used to verify the sequencing results.

### Statistical analysis

Results were presented as mean ± standard deviation. SPSS 14.0 software was used to analyze the significance using one-way ANOVA and two-way ANOVA. *P*<0.05 and *P*<0.01 were considered meaningful.

## Results

### Macrophages proliferation and viability

**Figure 2.**
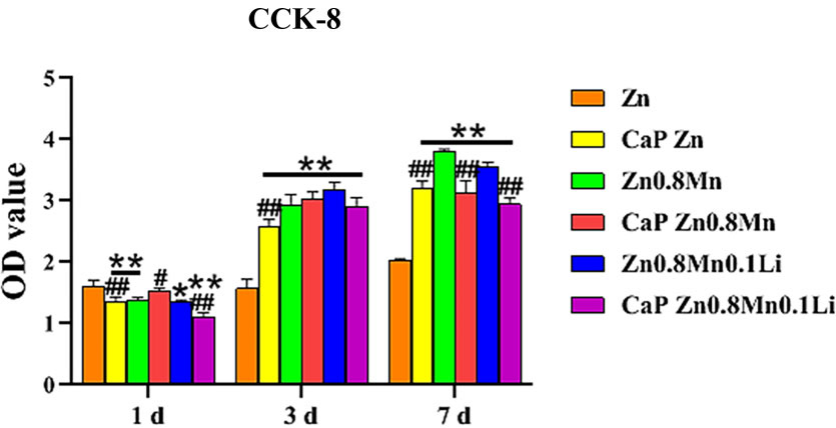
CCK-8 cells proliferation experiment. *P<0.05 vs Zn, **P < 0.01 vs Zn, ^##^*P* < 0.01 vs no coatings.

Using CCK-8 cell viability assay to determine activity after seeding macrophages cells on the surface of different samples for 1, 3 and 7 days, as shown in Fig. 2. The slow proliferation of macrophages on pure Zn scaffold was observed, while the proliferation of macrophages on Zn0.8Mn and Zn0.8Mn0.1Li scaffolds was significantly improved. Compared with uncoated scaffolds, the proliferative capacity of CaP Zn scaffold was improved. Macrophages on the surface of CaP Zn0.8Mn and CaP Zn0.8Mn0.1Li scaffold showed good proliferation ability, which were not significantly different from that of uncoated scaffolds.

**Figure 3.**
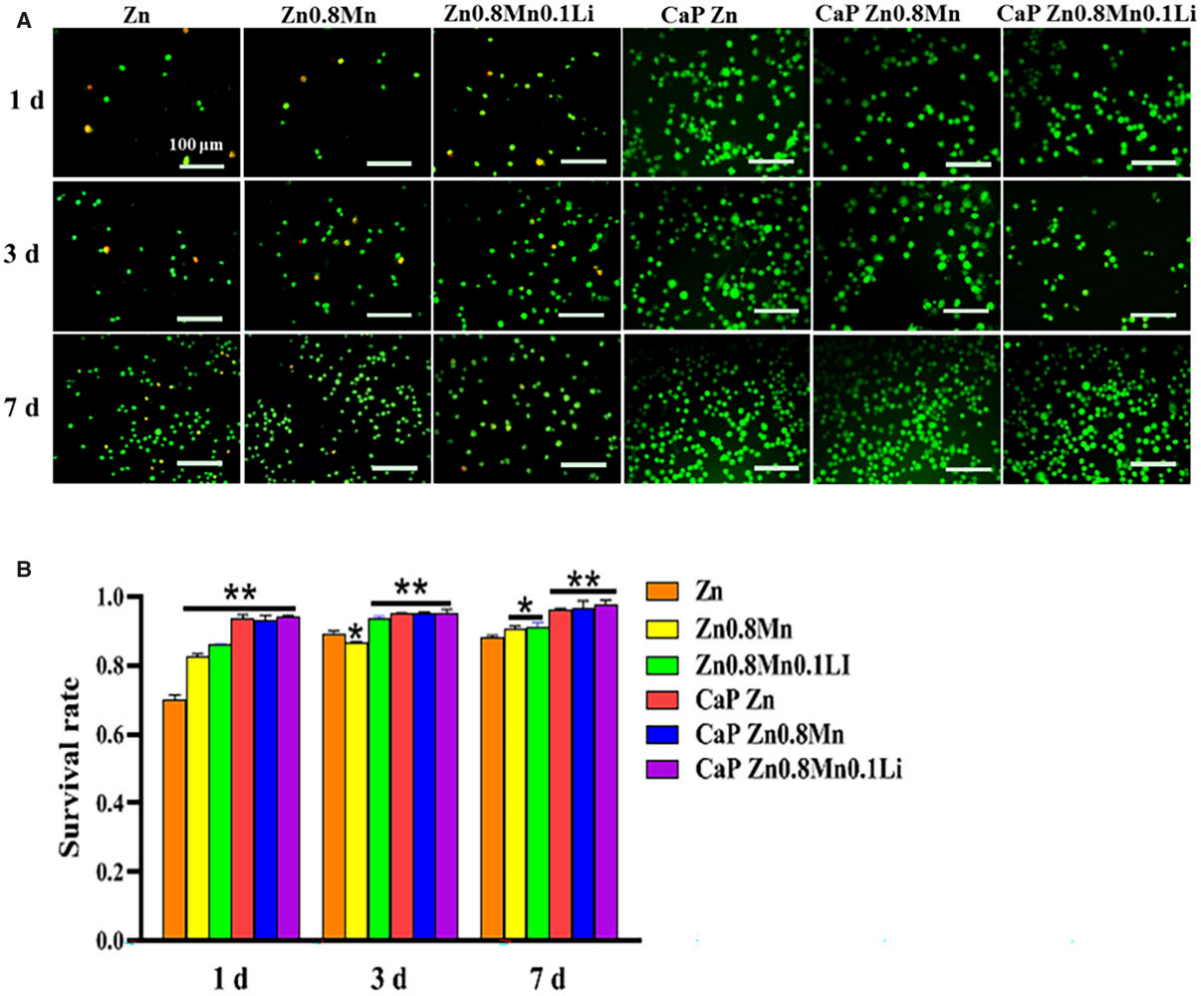
(**A**) AM/PI staining and (**B**) cell survival ratio. *P < 0.05 vs Zn, **P <0.01 vs Zn.

AM/PI staining was used to measure cell viability (Fig. 3A and B). These results indicated that the survival rate of Zn0.8Mn0.1Li scaffold group was higher than pure Zn and Zn0.8Mn scaffolds. No dead cells were observed on all CaP-coated Zn-Mn-Li scaffolds. In addition, the number of RAW264.7 macrophages on different surfaces increased linearly on the seventh day. These results indicated that Zn-Mn-Li scaffolds had good cytocompatibility with macrophages and can be used for subsequent studies on macrophages polarization.

### Morphological observation of macrophages

**Figure 4.**
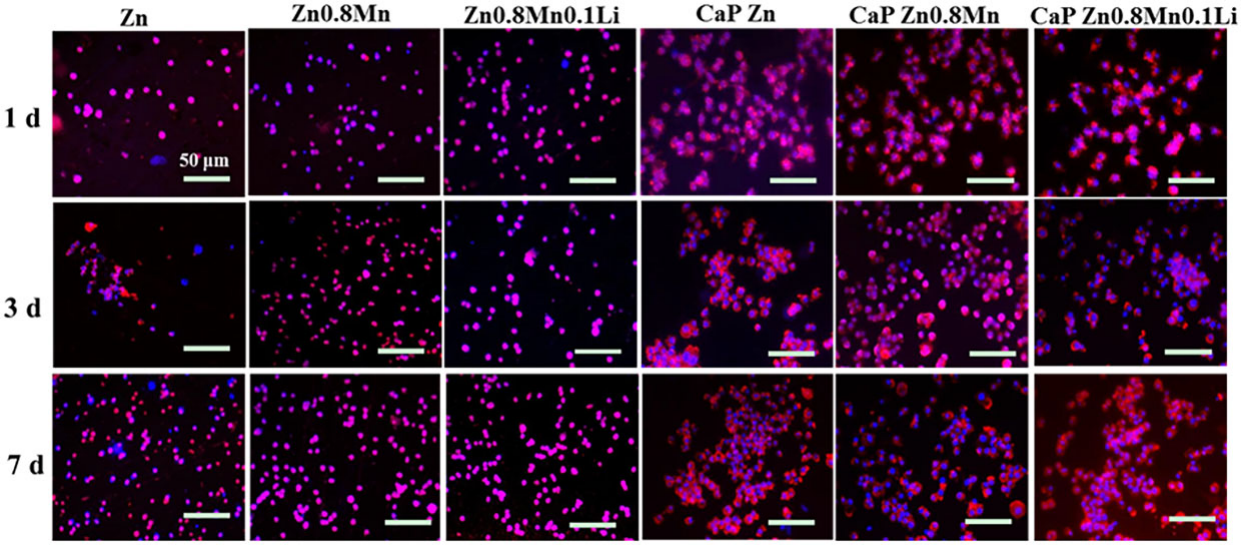
Cytoskeleton staining of RAW264.7.

The results of macrophages cytoskeleton staining were shown in Fig. 4. The macrophages on the surface of Zn, Zn0.8Mn and Zn0.8Mn0.1Li scaffolds were spherical, dispersed and not connected with other cells. After adding CaP coatings, the spreading area became larger and the cells were closely connected. The morphology of macrophages on the surface of CaP-coated Zn-Mn-Li scaffolds was similar.

**Figure 5.**
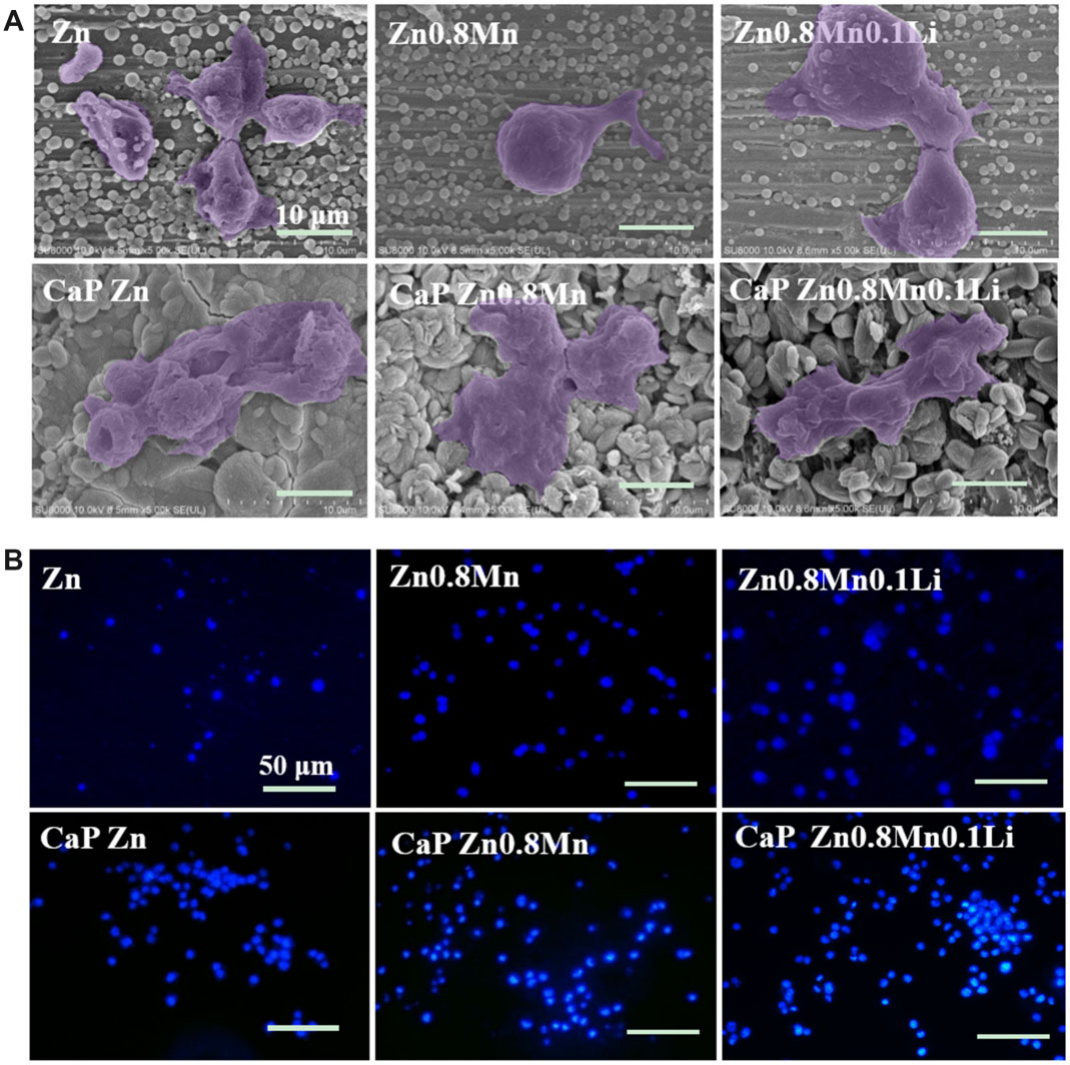
(**A**) SEM and (**B**) DAPI nuclear staining pictures of Raw264.7 cells on Zn-Mn-Li scaffolds surface after culturing 3 days.

The adhesion morphology of macrophages was shown in Fig. 5A. The macrophages on the surface of pure Zn were spherically small and shrunken to a certain extent. The macrophages on the surface of Zn0.8Mn and Zn0.8Mn0.1Li alloys scaffolds were mostly round with tiny pseudopods protrusion. The macrophages on the surface of the CaP Zn-Mn-Li alloys scaffolds were more full in shape, expanded and had pseudopodia protrudate, showing better adhesion ability. DAPI staining showed that the adhesion number of macrophages on the surface of Zn0.8Mn0.1Li scaffold was higher than that in pure Zn and Zn0.8Mn scaffolds. After the addition of CaP coatings, the number of macrophages adhesion increased, and the number of macrophages adhesion on CaP Zn0.8Mn0.1Li scaffold was the highest (Fig. 5B).

### Macrophages inflammatory response

**Figure 6.**
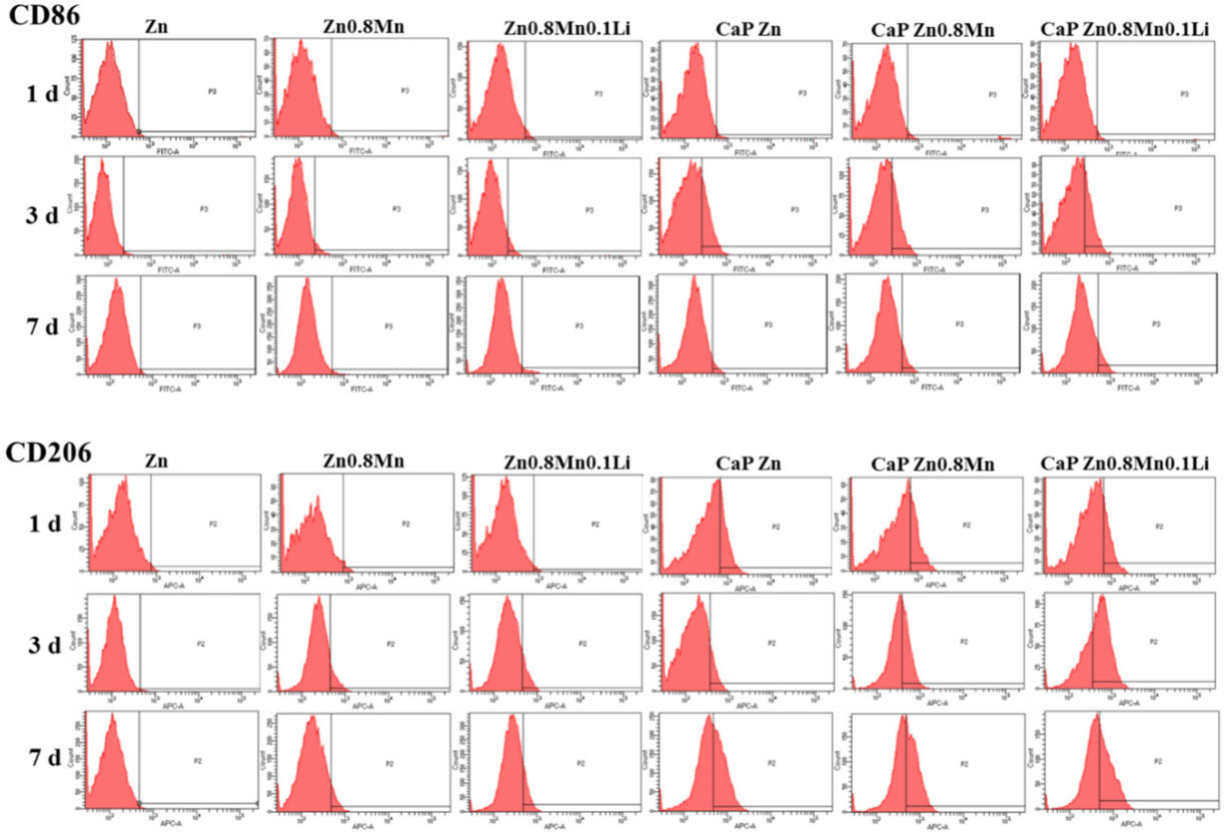
Percentages of CD86 or CD206-positive macrophages by flow cytometry.

The expressions of macrophages surface markers CD86 and CD206 were analyzed by flow cytometry. As shown in Fig. 6, the positive expressions of CD86 and CD206 were not obvious in macrophages on the surface of pure Zn, Zn0.8Mn and Zn0.8Mn0.1Li scaffolds. After CaP coatings were added, the positive expressions of CD86 were found on 3 days and significantly decreased on 7 days. CD206 was expressed on 1, 3 and 7 days and the expression of CD206 increased with the extension of time, indicating that CaP coatings can regulate the polarization of macrophages toward M2.

**Figure 7.**
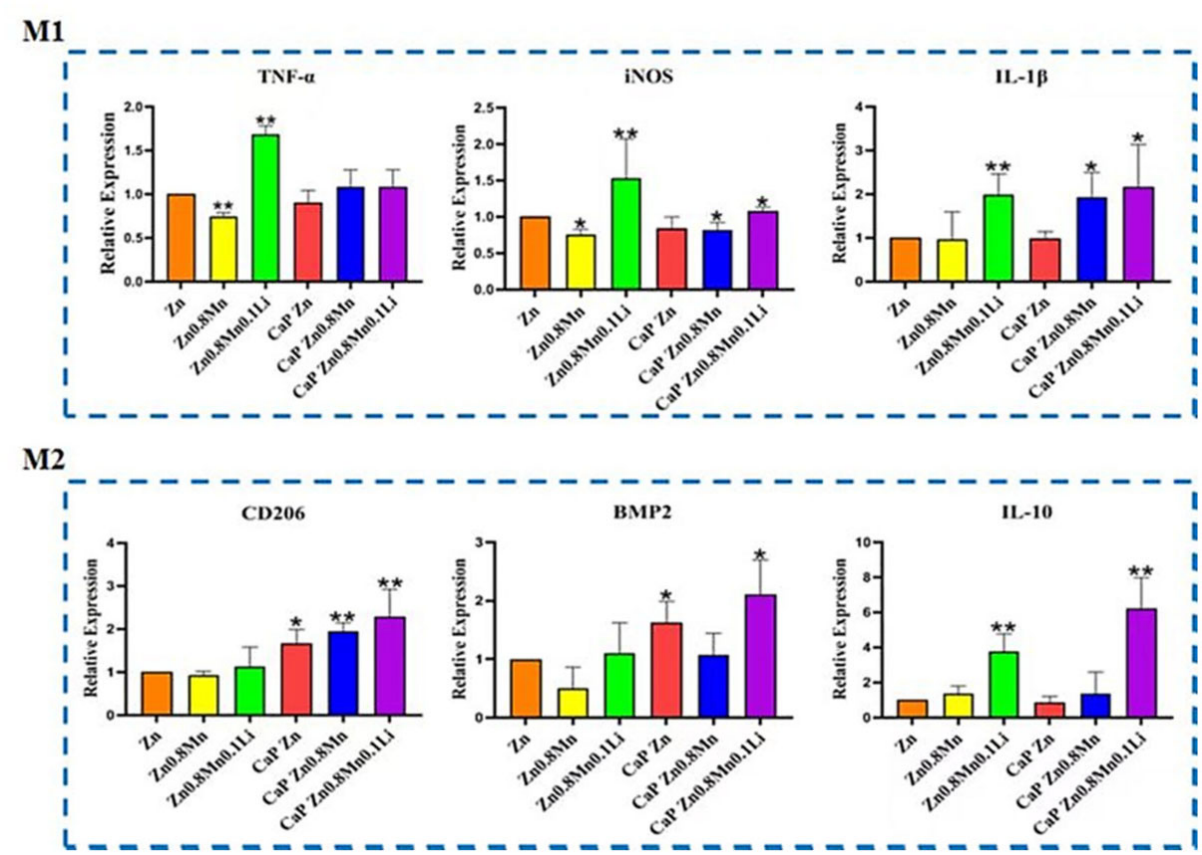
The expression of TNF-α, iNOS, IL-1β, CD206, BMP2 and IL-10 in macrophages after 3 days of culture on Zn-Mn-Li scaffolds. *P < 0.05 vs Zn, **P < 0.01 vs Zn.

The expression of polarization markers in macrophages was detected by qRT–PCR. As shown in Fig. 7, the expression of M1-type macrophage markers TNF-α, iNOS and IL-1β was not high on the coated Zn-Mn-Li scaffolds. Meanwhile, the expression of M2-type macrophages markers CD206, BMP2 and IL-10 on CaP Zn0.8Mn0.1Li scaffold was significantly enhanced, indicating that CaP Zn0.8Mn0.1Li scaffold could induce macrophages to be polarized toward M2.

### Osteogenic differentiation of MC3T3-E1 under the stimulation of the macrophages/scaffolds modulated osteo-immune environment

**Figure 8.**
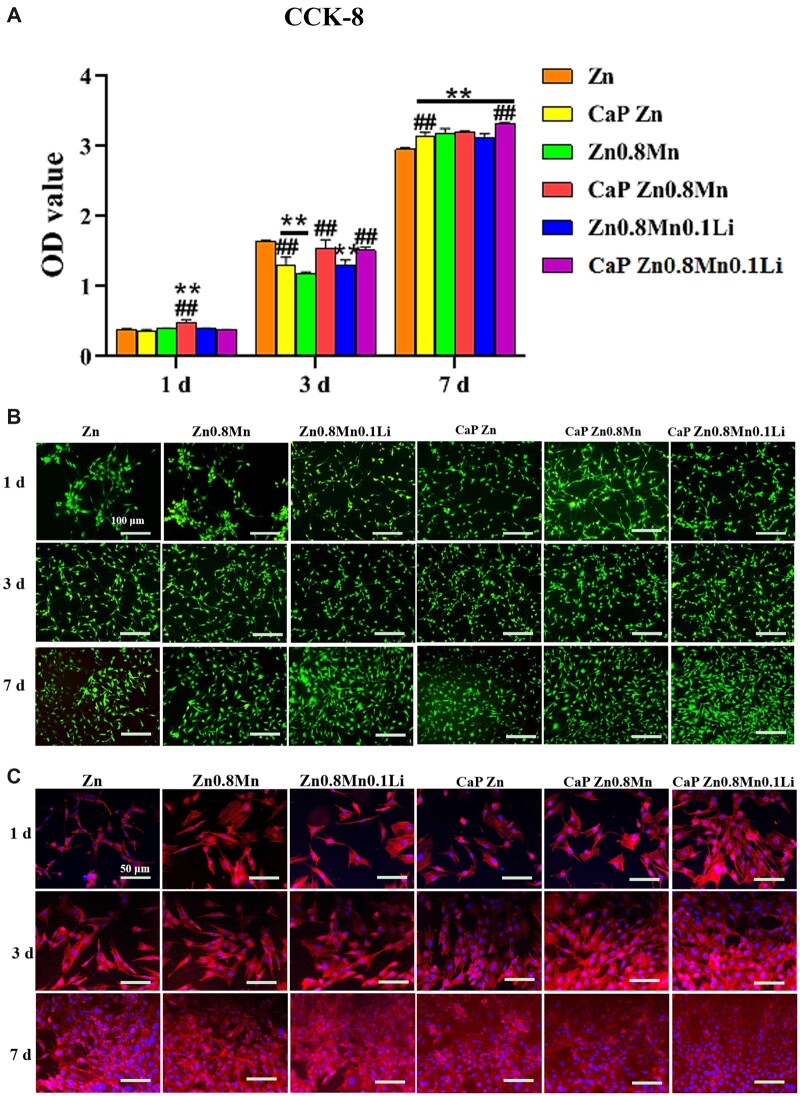
(**A**) CCK-8 cell proliferation experimentation, (**B**) AM/PI staining and (**C**) cytoskeleton staining. *P < 0.05 vs Zn, **P<0.01 vs Zn, ^##^*P* < 0.01 vs no coatings.

Based on the bone immune response of macrophages, we explored the effect of Zn-Mn-Li scaffolds/macrophages immune microenvironment on osteogenic differentiation of MC3T3-E1 cells. As shown in Fig. 8A, MC3T3-E1 cells in the pure Zn condition medium proliferated slowly, while the other groups had good proliferation effects. On Day 7, MC3T3-E1 cells in CaP Zn0.8Mn0.1Li scaffold CM had the highest proliferation. The staining results of living and dead cells were shown in Fig. 8B. The survival rate of each group was good with almost no dead cells. Phalloidin staining showed that the morphology of MC3T3-E1 cells in pure Zn CM was affected on the first day, the cells became thinner and the spread area was smaller. MC3T3-E1 cells showed better morphological spread in Zn0.8Mn, Zn0.8Mn0.1Li and CaP Zn CM, and the spread area of MC3T3-E1 cells was larger than that in pure Zn group. MC3T3-E1 cells were polygonal in CaP Zn0.8Mn and CaP Zn0.8Mn0.1Li CM and fusiform microfilaments spread throughout the whole cell and extended in all directions. It was proved that the addition of Mn/Li element and the preparation of CaP coatings can promote the cytoskeletal rearrangement and cell spreading of MC3T3-E1 (Fig. 8C).

**Figure 9.**
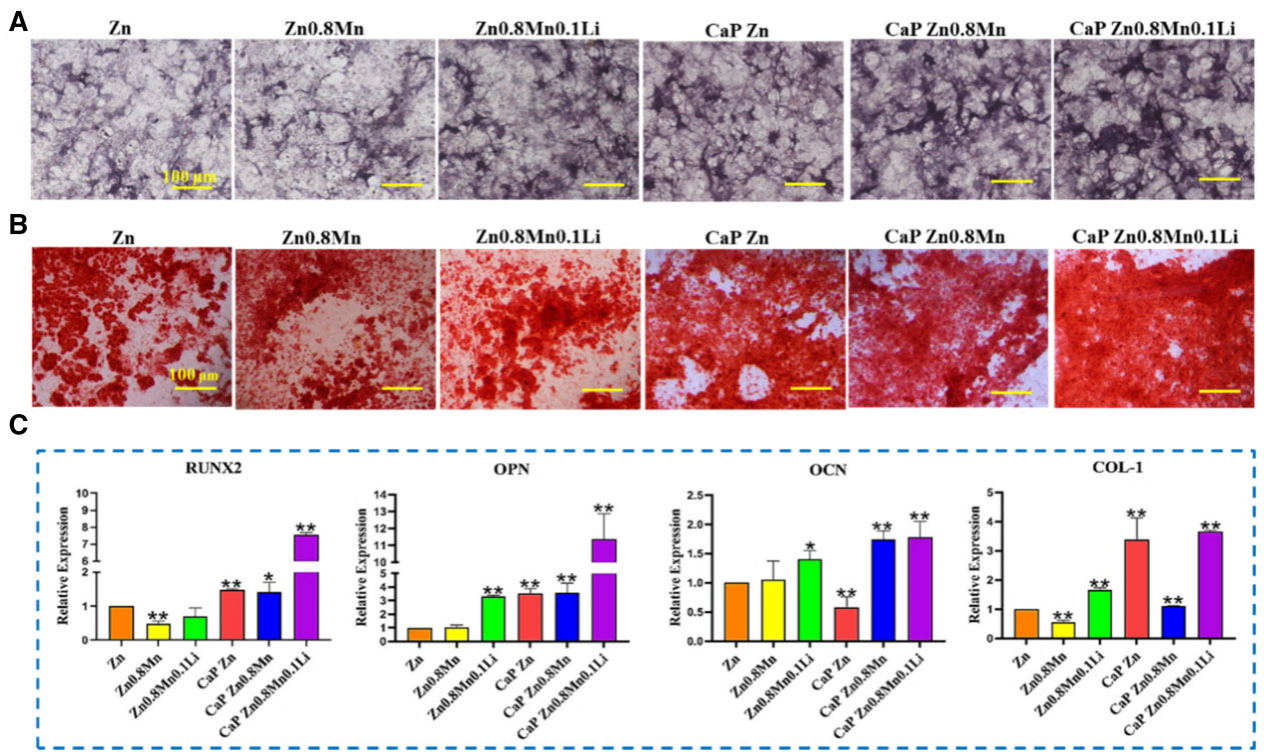
(**A**) ALP staining, (**B**) Alizarin red staining and (**C**) gene expression of osteogenesis-related markers detected by qRT–PCR. *P < 0.05 vs Zn, **P < 0.01 vs Zn.

The osteogenic ability of MC3T3-E1 cells was detected by ALP staining, alizarin red staining and the expression of osteogenic genes. As shown in Fig. 9, CaP Zn0.8Mn0.1Li scaffold had the highest positive expression of ALP and the highest number of Ca nodules (Fig. 9A and B). Meanwhile, MC3T3-E1 cells stimulated with macrophages/CaP Zn0.8Mn0.1Li scaffold CM had the highest expression of osteogenic genes (RUNX2, OPN and OCN, COL-1) (Fig. 9C). Therefore, we speculated that CaP Zn0.8Mn0.1Li scaffold can induce M2-type polarization of macrophages, which can better stimulate the inflammatory response of the body and accelerate bone reconstruction. In response to CaP Zn0.8Mn0.1Li scaffold, M2 macrophages generated the bone immune microenvironment that effectively promoted bone regeneration.

### Analysis of osteogenic ability *in vivo*

**Figure 10.**
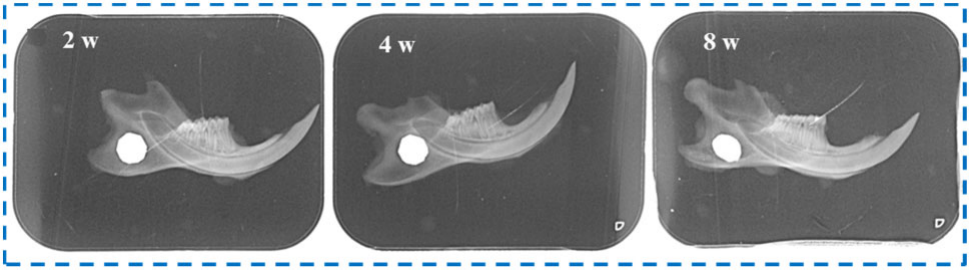
X-ray observation of mandible defect implanted with Zn0.8Mn0.1Li scaffold.

The implantation of Zn-Mn-Li scaffolds in rats was observed by X-ray. The results showed that the defect was located in front of the mandibular angle, and no nerve injury and no fracture occurred. At 2 weeks, there was a clear boundary between the scaffolds and normal bone tissue, and the new bone formation was not obvious. At 4 weeks, the X-ray showed that the material was degraded and the defect area was significantly reduced. At 8 weeks, the CaP Zn0.8Mn0.1Li scaffold was obviously degraded, and there was no gap around the defect, indicating that the defect area was reduced and new bone formed (Fig. 10).

**Figure 11.**
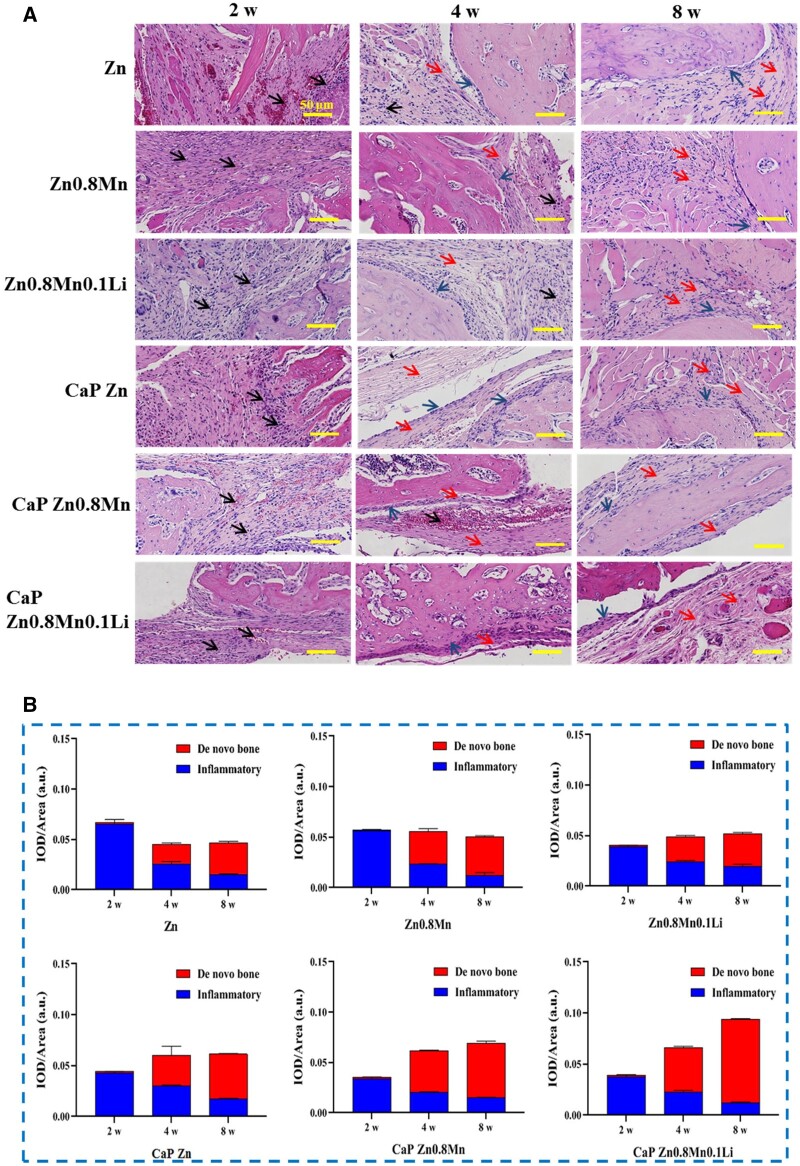
(**A**) Histomorphological analysis by H&E staining (black arrow: inflammatory cells, red arrow: new bone matrix, blue arrow: osteoblasts) and (**B**) semi quantification of inflammatory and *de novo* bone area.

H&E results showed that Zn-Mn-Li scaffolds affected cell infiltration and formation of new bone matrix. At 2 weeks, a lot of inflammatory cells appeared in the defect site, mainly manifested as inflammation, and no obvious new bone formation was observed around the implantation. At 4 weeks, osteoblasts were observed to be distributed in strips along the defect edge, with new bone matrix and fibrous tissue surrounding the materials. At 8 weeks, a large number of new bone matrix was formed, in which CaP Zn0.8Mn0.1Li scaffold showed weak inflammatory manifestations and the most new bone matrix (Fig. 11A). The results of semi-quantitative analysis of inflammation and new bone matrix were shown in Fig. 11B. At 2 weeks after surgery, inflammatory reactions occurred in all groups, and there was basically no new bone matrix formation. At 4 and 8 weeks, inflammation decreased in all groups, and the area of new bone matrix gradually increased. The matrix area of new bone in CaP Zn0.8Mn0.1Li group was significantly higher than that in other groups at 4 and 8 weeks, and the inflammatory reaction of CaP Zn0.8Mn0.1Li group was the lowest.

**Figure 12.**
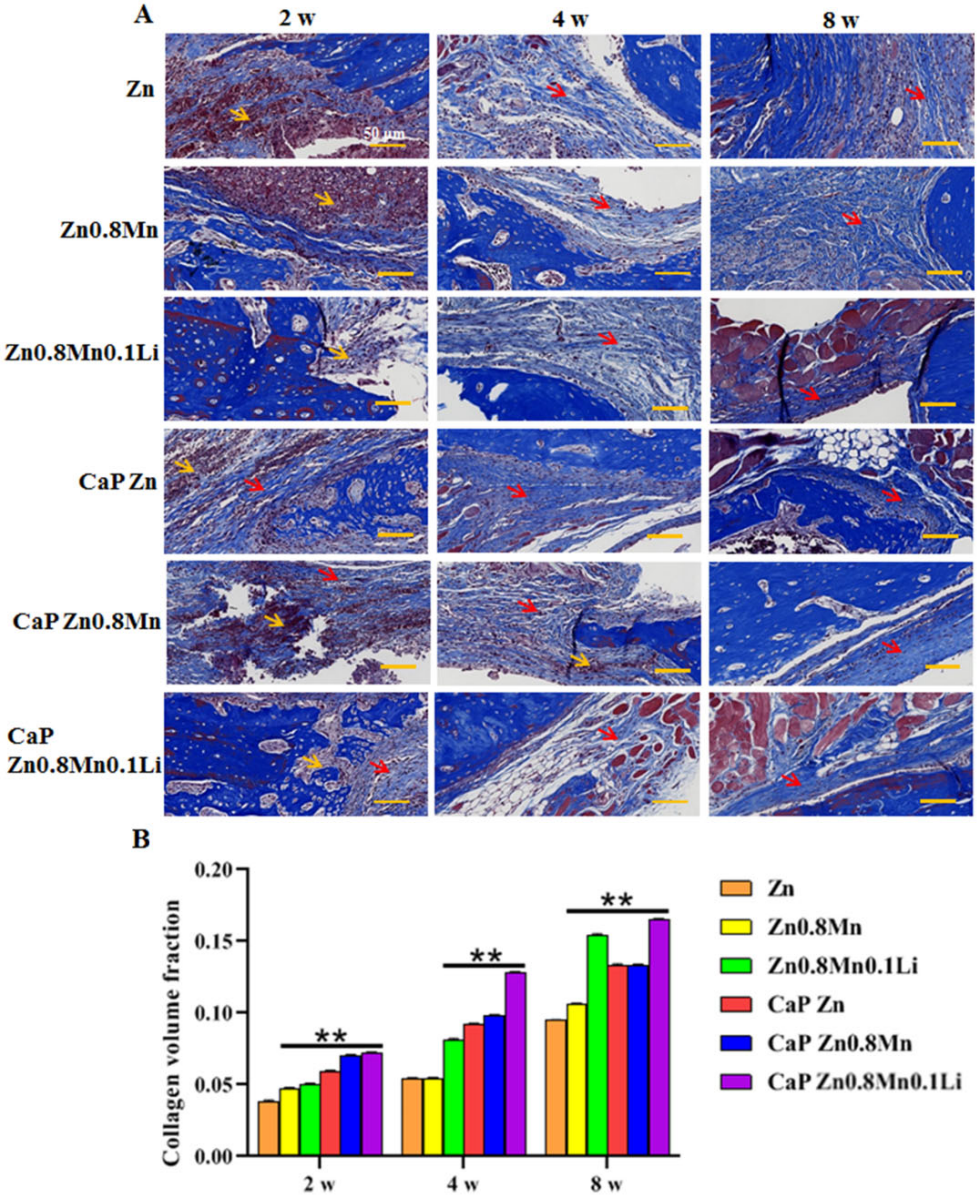
(**A**) Masson’s staining of bone tissue (red arrow: new bone matrix, yellow arrow: necrotic cells) and (**B**) semi quantification of *de novo* bone area. **P < 0.01 vs Zn.

In Masson staining, collagen fibers were dyed blue, new bone fibers were dyed light blue and muscle fibers and red blood cells were dyed red (Fig. 12A). At 2 weeks, compared with the CaP Zn0.8Mn0.1Li scaffold, there were a large number of necrotic cells, inflammatory reactions and few new bone fibers in the other groups. With the extension of time, the scaffolds gradually degraded, and new collagen fibers and the connection between the host and the defect formed gradually. Compared with other scaffolds, the CaP Zn0.8Mn0.1Li scaffold demonstrated better bone regeneration, characterized by infiltration of new bone fibers and stroma and cells, and the emergence of a large number of repaired tissues (Fig. 12B). This suggested that the CaP Zn0.8Mn0.1Li scaffold can provide the favorable microenvironment for bone tissue growth.

### RNA sequencing analysis of macrophages

**Figure 13.**
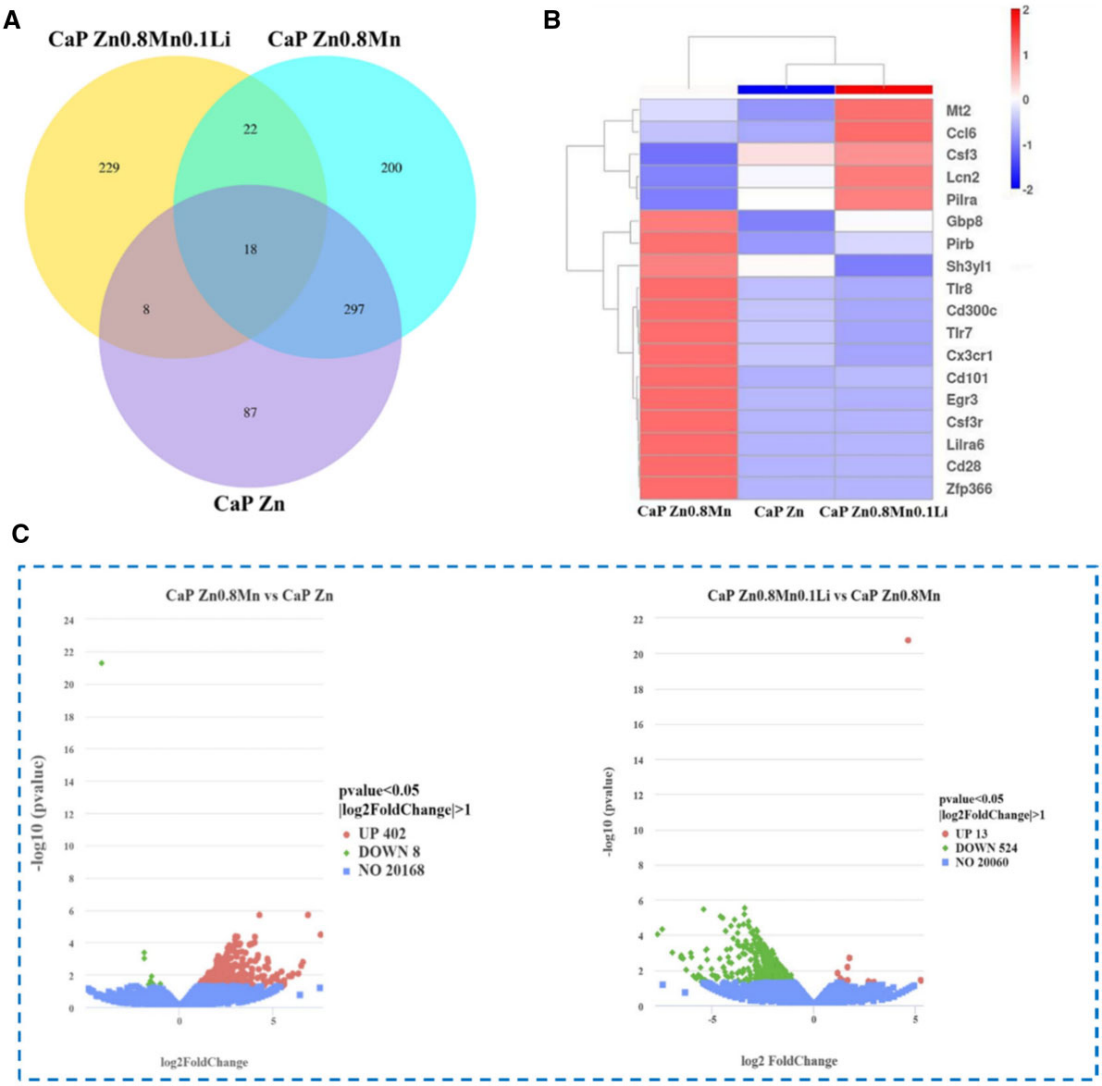
(**A**) Venn plot among the groups, (**B**) volcano map of differentially expressed genes and (**C**) heatmap of the distinct upregulated and downregulated genes.

**Figure 14.**
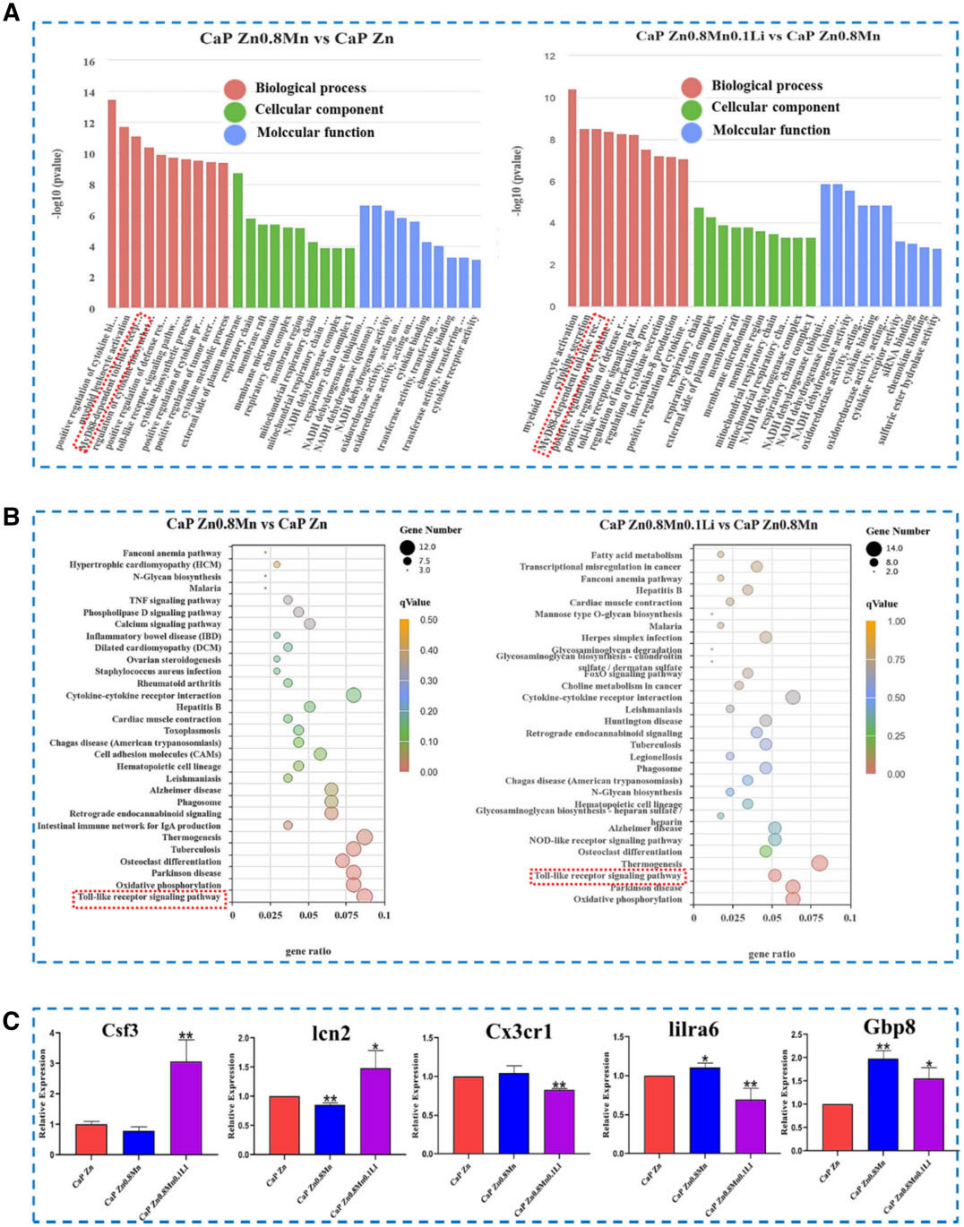
(**A**) GO analysis of differential gene expression, (**B**) KEGG pathway analysis and (**C**) RT–PCR results for Csf3, lcn2, Cx3cr1, lilra6 and Gbp8, respectively. *P < 0.05 vs Zn, **P < 0.01 vs Zn.

Macrophages were cultured on CaP-coated Zn-Mn-Li alloys for 3 days for RNA sequencing. Venn diagram of gene expression showed that there were 87 specific genes in the CaP-Zn group and 200 specific genes in the CaP-Zn0.8Mn group. There were 229 specific genes in CaP Zn0.8Mn0.1Li group (Fig. 13A). In Fig. 13B, it showed that CaP Zn0.8Mn upregulated 402 genes and downregulated 8 genes compared with CaP Zn. Meanwhile, compared with CaP Zn0.8Mn, CaP Zn0.8Mn0.1Li upregulated 13 genes and downregulated 524 genes. Cluster analysis heat map screened out the genes of CaP Zn0.8Mn0.1Li scaffold that showed differences compared with the other two groups (*P*-values <0.05), which included genes associated with immune processes, such as Csf3, Lcn2 and Pirb (Fig. 13C). CaP Zn0.8Mn and CaP Zn0.8Mn0.1Li participated in the same biological processes, mainly including the myeloid leukocyte activation, regulation of cytokine biosynthetic process, positive regulation of defense response, Toll-like receptor signaling pathway, cytokine metabolic process, external side of plasma membrane, NADH dehydrogenase activity, oxidoreductase activity, cytokine binding, chemokine binding and so on (Fig. 14A). According to KEGG signaling pathway analysis, CaP Zn0.8Mn0.1Li was significantly enriched in several signaling pathways related to macrophages polarization, especially Toll-like receptor signaling pathways (Fig. 14B). Realtime-PCR was used to verify related differential genes, and the results were identical to transcriptome sequencing (Fig. 14C).

## Discussion

It is well known that Zn is an essential nutrient element for human body, involved in nucleic acid metabolism, signal transduction, cell apoptosis and gene expression [19, 20]. However, the mechanical properties of pure Zn cannot meet the applications in clinical medical materials, and further improvement is needed to meet the clinical needs. In order to ensure the biosafety of implant materials and take skeletal applications into account, nutrient elements Mn and Li, which are closely related to bone tissue, are selected as alloying elements to make up for the lack of mechanical properties of pure Zn through alloying [21, 22]. Previous research results showed that after the addition of Mn and Li element, the second phase of MnZn_13_ and LiZn_4_ appeared, which resulted in grain refinement and hardness improvement. At the same time, the degradation rate was effectively regulated, avoiding the cytotoxicity caused by excessive local Zn^2+^ concentration [2].

Surface modification is an effective method to obtain specific material properties and promote bone integration [23]. Phosphate chemical conversion technology is an effective technology to form conversion films containing different elements and different types on the surface by controlling morphology and element composition. Previous studies had shown that CaP coatings have good physical and chemical properties and *in vitro* osteogenic potential [24–26]. Crosstalk between immune cells and bone cells is crucial for regulating inflammation and initiating new bone formation [27]. This study showed that osteogenic differentiation of osteoblasts was not only dependent on the properties of biomaterials, but also influenced by the immune microenvironment formed by the interaction between immune cells and biomaterials. Therefore, creating a bone immune microenvironment that stimulates osteogenesis through further optimized design of biomaterials is essential for bone tissue regeneration and repair.

Macrophages are very sensitive to the physical and chemical properties of biomaterials and different surface properties transmit different polarization signals to the macrophages. M1-type macrophages and M2-type macrophages can mediate different host immune response to implants and play different roles in bone regeneration and repair. In the early stage of implantation, M1-type macrophages play a pro-inflammatory function, phagocytic cell fragments and dead neutrophils, secrete inflammatory cytokines, such as TNF-α, IL-1β and IL-6, and regulate the recruitment and migration of macrophages. At the later stage of implantation, M2-type macrophages produce a large number of osteogenic, angiogenic and anti-inflammatory cytokines, including BMP2, CD206 and IL-10, which reduce inflammatory response and accelerate bone repair [18, 28, 29]. The results on the polarization of macrophages showed that the CaP Zn0.8Mn0.1Li scaffold could induce the polarization of macrophages toward M2. M2-type macrophages secreted a series of chemokines and growth factors related to tissue regeneration to promote extracellular matrix remodeling and tissue healing, playing an important role in bone regeneration and repair.

The surface structure and morphology of the biomaterials affect the adhesion ability and further affect inflammation and fibrosis [30]. The physical and chemical properties of biomaterials can regulate the shape of macrophages and further affect the polarization and function [31–33]. Our study proved that compared with osteoblasts, no highly ordered bundled cytoskeleton structure was found in RAW264.7 cells. The macrophages on the surface of Zn, Zn0.8Mn and Zn0.8Mn0.1Li alloys were spherical, dispersed and unconnected with other cells. After the addition of CaP coatings, the spread area of macrophages was enlarged and the cells were closely connected. The adhesion number of macrophages on the surface of CaP Zn0.8Mn0.1Li alloy was the highest. The polarization trend of macrophages on the surface of the scaffold containing Li element was more obvious, indicating that Li element stimulated macrophages more significantly. CaP coatings can activate macrophages, and the addition of CaP coatings can stimulate the polarization of macrophages toward M2 direction, and the polarization level of CaP Zn0.8Mn0.1Li group was the most obvious. In the study, we demonstrated that the synergistic action of CaP coatings with Mn/Li elements can stimulate macrophages polarization toward M2. The CaP Zn0.8Mn0.1Li alloy scaffold can regulate the polarization of macrophages toward M2, generate an immune microenvironment conducive to bone regeneration and effectively accelerate bone integration.

Crosstalk between immune cells and bone cells was essential for regulating inflammation and initiating new bone formation [34]. Osteogenic differentiation of osteoblasts depended not only on the properties of biomaterials but also on the immune microenvironment formed by the interaction between immune cells and biomaterials [35]. CaP Zn0.8Mn0.1Li scaffold could induce the polarization of macrophages toward M2, inhibit the inflammatory response and promote M2-type macrophage mediated osteogenic differentiation. Specifically, the CaP Zn0.8Mn0.1Li scaffold induced macrophages to transition to the M2 phenotype, reduced inflammatory responses and released bone-inducing molecules, thereby promoted osteogenic differentiation of MC3T3-E1 cells, which may be related to toll-like receptor signaling pathways.

**Figure 15.**
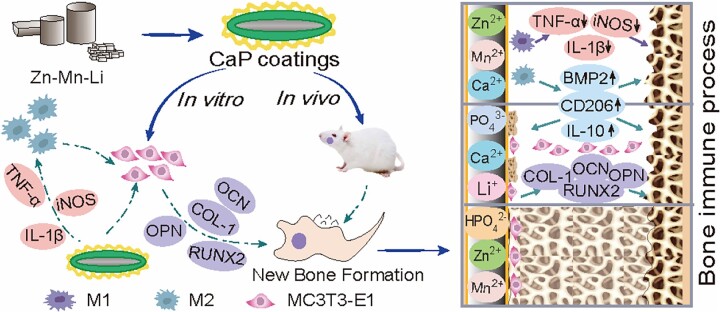
Schematic diagram of bone immune regulation of Zn-Mn-Li scaffolds.

In order to analyze the effect of CaP Zn-Mn-Li alloys *in vivo*, Zn-Mn-Li scaffolds were implanted into the mandible of rats. The results showed that CaP Zn0.8Mn0.1Li scaffold could well induce new bone formation, suggesting that CaP coatings may be an effective Zn-Mn-Li scaffolds by inducing the polarization of M2 macrophages to reduce inflammatory damage of surrounding tissues and accelerate bone repair. At the same time, the importance of considering the immune response when evaluating the osteogenic ability of bone replacement materials was further emphasized. Zn-Mn-Li scaffolds can regulate the local immune microenvironment and have a significant impact on the function of osteoblasts, thus affecting bone regeneration and repair [36, 37]. In this study, we demonstrated that CaP coatings prepared on Zn-Mn-Li scaffolds can effectively regulate bone immune performance, making it more conducive to bone formation (Fig. 15).

Macrophages polarization played an important role in bone remodeling and played a decisive role in bone regeneration around the implant [38]. M2 macrophages were involved in anti-inflammation, tissue healing and angiogenesis [39]. Regulating M2 polarization of macrophages had become a new strategy in tissue engineering. In order to understand the mechanism of CaP Zn0.8Mn0.1Li scaffold induced macrophages polarization to promote bone integration, we used RNA sequencing technology to explore the changes of macrophages signaling pathway after the addition of Mn and Li. The results showed that CaP Zn0.8Mn0.1Li scaffold was involved in the secretion of inflammatory cytokines, affected cell metabolism and regulated transmembrane signal receptor communication. Furthermore, the genes involved in these pathways were further grouped to study their roles in bone integration. The results showed that CaP Zn0.8Mn0.1Li scaffold could significantly activate Toll-like signaling pathway, inhibited inflammatory response and accelerated bone integration after addition of Mn and Li elements. Follow-up studies will be conducted to uncover the potential mechanism by which CaP Zn0.8Mn0.1Li scaffold regulated macrophage polarization and accelerated bone integration.

## Conclusion

Modified Zn-Mn-Li alloys with CaP coatings changed the harmful immunomodulatory properties of bone and made them more favorable to bone integration. Specifically, the CaP Zn0.8Mn0.1Li alloy induced an effective transformation of the M2 macrophages phenotype, inhibited inflammation and shifted the immune microenvironment to one that favored osteogenesis rather than osteoclasts, and enhanced the osteogenic differentiation of MC3T3-E1 cells. The underlying mechanism of this process may be related to Toll-like receptor signaling pathways. Therefore, the preparation of CaP coatings on the surface of the Zn-Mn-Li alloys surface is a valuable method to promote bone integration. In addition, implanting bone biomaterials with good bone immunomodulatory properties is a valuable strategy for developing advanced bone biomaterials.

## Data Availability

The raw data supporting the conclusion of this article will be made available by the authors, without undue reservation.
